# Optimal surveillance of intraductal papillary mucinous neoplasms of the pancreas focusing on remnant pancreas recurrence after surgical resection

**DOI:** 10.1186/s12885-022-09650-w

**Published:** 2022-05-29

**Authors:** Tomokazu Fuji, Yuzo Umeda, Kosei Takagi, Ryuichi Yoshida, Kazuhiro Yoshida, Kazuya Yasui, Kazuyuki Matsumoto, Hironari Kato, Takahito Yagi, Toshiyoshi Fujiwara

**Affiliations:** 1 Department of Gastroenterological Surgery, Okayama University Graduate School of Medicine Dentistry and Pharmaceutical Sciences, 2-5-1 Shikata-cho, Okayama, 700-8558 Japan; 2 Department of Gastroenterology and Hepatology, Okayama University Graduate School of Medicine Dentistry and Pharmaceutical Sciences, Okayama, Japan

**Keywords:** Pancreatic intraductal neoplasms, Pancreatectomy, Recurrence

## Abstract

**Background:**

The international consensus guidelines for intraductal papillary mucinous neoplasm of the pancreas (IPMN) presented clinical features as indications for surgery. Whereas surveillance for recurrence, including de novo lesions, is essential, optimal surveillance protocols have not been established.

**Aim and methods:**

This study aimed to assess the clinical features of recurrence at the remnant pancreas (Rem-Panc) and extra-pancreas (Ex-Panc) after surgery for IPMN. Ninety-one patients of IPMN that underwent detailed preoperative assessment and pancreatectomy were retrospectively analyzed, focusing especially on the type of recurrence.

**Results:**

The IPMNs were finally diagnosed as low-grade dysplasia (LDA, *n* = 42), high-grade dysplasia (HAD, *n* = 19), and invasive carcinoma (IPMC, *n* = 30). Recurrence was observed in 26 patients (29%), of which recurrence was seen at Rem-Panc in 19 patients (21%) and Ex-Panc in 7 patients (8%). The frequency of Rem-Panc recurrence was 10% in LDA, 21% in HDA, and 37% in IPMC. On the other hand, Ex-Panc recurrence was observed only in IPMC (23%). Ex-Panc recurrence showed shorter median recurrence-free survival (RFS) and overall survival (OS) than Rem-Panc recurrence (median RFS 8 months vs. 35 months, *p* < 0.001; median OS 25 months vs. 72 months, *p* < 0.001). Regarding treatment for Rem-Panc recurrence, repeat pancreatectomy resulted in better OS than no repeat pancreatectomy (MST 36 months vs. 15.5 months, *p* = 0.033). On multivariate analysis, main duct stenosis or disruption as a preoperative feature (hazard ratio [HR] 10.6, *p* = 0.002) and positive surgical margin (HR 4.4, *p* = 0.018) were identified as risk factors for Rem-Panc recurrence.

**Conclusions:**

The risk factors for Rem-Panc and Ex-Panc recurrence differ. Therefore, optimal surveillance on these features is desirable to ensure that repeat pancreatectomy for Rem-Panc recurrence can be an appropriate surgical intervention.

**Supplementary Information:**

The online version contains supplementary material available at 10.1186/s12885-022-09650-w.

## Introduction

For the clinical management of intraductal papillary mucinous neoplasms of the pancreas (IPMN), an international consensus guideline was provided by the International Association of Pancreatology [1–3]. These guidelines for clinical management, including radiological and endoscopic follow-up and surgical indications, have been widely accepted. After surgical resection, the postoperative recurrence rate of IPMN was reported to be 6.8 to 9.6% with non-invasive IPMN and 32.2 to 65% with invasive IPMN [4–7]. Notably, IPMN recurrences have various temporospatial distributions [7]. Previous studies of postoperative recurrence sites of IPMN showed that distant metastasis occurred mostly in invasive IPMN. The form of recurrence depends on the differentiation of the primary IPMN; the recurrence rates were 0-7.7% with remnant pancreas (Rem-Panc) recurrence, 0-1.3% with extra-pancreatic (Ex-Panc) metastasis, and 0-0.8% with both sites in non-invasive IPMN, whereas they were 5.7-15.0%, 35.0-45.7%, and 2.9-8.8% in invasive cancer [4, 5, 8]. Perioperative risk factors for the recurrence pattern have been debated. Risk factors for Rem-Panc recurrence were reported to be the presence of high-grade dysplasia in resected specimens, positive surgical margins, and a family history of pancreatic ductal adenocarcinoma (PDAC). It was also suggested that the gastric or pancreatobiliary type as IPMN subtypes could be potential indicators of carcinogenic recurrence as PDAC in Rem-Panc [9, 10].

Several guidelines for IPMN refer to the risk of postoperative recurrence and the follow-up policy. For non-invasive IPMN, including low-grade and high-grade dysplasia, surveillance with a focus on the remnant pancreas is recommended because early detection of a malignant lesion and surgical resection may improve the prognosis. The International Consensus Guideline 2017 advocates that cross-sectional imaging be required for high-risk factors for Rem-Panc recurrence, such as a family history of pancreatic cancer; non-invasive IPMN or IPMN-associated invasive carcinoma (IPMC) at the surgical margin; and non-intestinal subtypes [3]. However, in reality, the first two are relatively rare. In addition, the European Study Group On Cystic Tumors of The Pancreas guidelines specify high-grade dysplasia and main duct type as high-risk factors [11], and the AGA guidelines do not recommend periodic screening for IPMN low-grade dysplasia (LGD) because of its lack of cost-effectiveness [12]. Thus, there has not been a consensus in this field. From these points of view, this study aimed to assess the clinical features of IPMN recurrence.

## Materials and methods

### Study subjects

A total of 91 patients of IPMN that underwent initial surgical resection with curative intent in Okayama University Hospital from May 2007 to December 2014 were retrospectively reviewed. In addition, demographic information, symptoms at presentation, radiological and endoscopic findings, surgical procedures, pathology, and postoperative course were collected from medical records.

With regard to precise preoperative tumor assessment, all patients were assessed by endoscopic ultrasonography (EUS) using contrast enhancement for intraductal mural nodules and pancreatic juice cytology under endoscopic retrograde cholangiopancreatography (ERCP). In addition, the main pancreatic duct diameter or size of the branched cyst was measured by computed tomography (CT) or MRI. These diagnostic modalities determined definite mural nodules or pancreatic duct features such as stenosis or disruption. Preoperative pancreatitis and obstructive jaundice were judged by clinical findings including laboratory or radiological imaging data.

### Surgical indications, pathological examination, and postoperative follow-up

Indications for surgery were determined on the basis of international consensus guidelines in 2006 [1] or 2012 [2]. Patients with a preoperative diagnosis or highly suspicious of invasive cancer underwent pancreatectomy with regional lymph node dissection. Resected specimens were reviewed by pathologists and classified into three groups according to the World Health Organization (WHO) classification: IPMN low-grade dysplasia (LGD), IPMN high-grade dysplasia (HGD), and IPMN-associated invasive carcinoma (IPMC). If different grades coexisted in one lesion, the highest degree of dysplasia was adopted as the classification. Surgical margins were examined at pancreatic transection margins and dissected peripancreatic tissue margins, which were classified as negative or positive for LGD, HGD, and IPMC. The final staging was based on these findings and the TNM classification in the seventh edition [13].

For six months, adjuvant chemotherapy by gemcitabine or a fluorouracil-based agent was given to the patients with a final pathological diagnosis of IPMC. Postoperative follow-up with CT or MRI was performed according to the final pathology. Follow-up intervals varied by IPMN-classification: 6 to 12 months for LGD, 3 to 6 months for HGD, and three months for IPMC. If suspected tumor recurrence or metastasis was suspected, further examinations, including EUS, CT, and MRI, were performed as needed.

Tumor recurrence was classified into two types. One type was Rem-Panc recurrence, including de novo IPMN, PDAC, and obvious enlargement of preexisting IPMN in the remnant pancreas. The diagnosis of Rem-Panc recurrence required an endoscopic biopsy. The other type was extrapancreatic (Ex-Panc) recurrence, including metastases to the liver, lung, lymph node, and peritoneum (Supplemental Fig. 1).

### Statistical analysis

Clinical variables were compared using the Mann-Whitney U test for continuous data and Pearson’s correlation coefficient for categorical data. Continuous variables are presented as medians and interquartile range (IQR). Values of *p* < 0.05 were considered significant. Overall survival (OS), recurrence-free survival (RFS), and incidence of Rem-Panc recurrence were evaluated using the Kaplan-Meier method and compared using log-rank tests. Cox’s proportional hazards model with clinical variables including high-risk stigmata and worrisome EUS features [2] was used to identify prognostic factors for Rem-Panc recurrence. For this analysis, clinical variables showing values of *p* < 0.05 on univariate analyses were entered into the multivariate analysis. The event of Ex-Panc recurrence and other causes of death were treated as censored. Hazard ratios (HRs) and 95% confidence intervals (95%CIs) were calculated. All statistical analyses were performed using JMP version 14 (SAS Institute Inc., Cary, NC, USA).

### Ethics approval and consent to participate

This study conformed to the Declaration of Helsinki on Human Research Ethics standards and was approved by the Okayama University Hospital Institutional Ethics Board (number 1902–019). The need for written, informed consent was waived by the Okayama University Hospital Institutional Ethics Board because of the retrospective design.

## Results

The enrolled patients' clinical and pathological background characteristics are summarized in Table 1. As preoperative examinations, contrast-enhanced CT was performed in all patients, EUS in 89 patients (97.8%), and pancreatic juice cytology in 78 patients (85.7%). The most frequent pathological type was LGD (*n* = 42, 46.2%), followed by IPMC (*n* = 30, 33.0%) and HGD (*n* = 19, 20.9%). Sixty patients (65.9%) had negative surgical margins; the other 31 patients had positive surgical margins. These surgical margins were pathologically diagnosed as LGD (*n* = 22, 24.2%), HGD (*n* = 6, 6.6%), and IPMC (*n* = 3, 3.3%). In three patients with positive margins as IPMC, postoperative diagnosis overturned the result, despite a negative margin on intra-operative frozen section examination. There was no hospital mortality. Twenty patients received adjuvant chemotherapy based on a final pathology of IPMC with T3-4 or positive lymph node; one patient was diagnosed as stage 1A, and the other 19 patients were as UICC-stage 2A/2B. The median follow-up periods after resection were 71 months (IQR, 37-103 months). In this follow-up period, tumor recurrence was confirmed in 26 patients (28.6%), of whom 19 patients (20.9%) showed Rem-Panc recurrence, and seven patients (7.7%) showed Ex-Panc recurrence (Supplementary Fig. 1).

**Table 1:** Summary of clinicopathological characteristics and correlations between recurrence patterns

	All	no recurrence	Rem-Panc	Ex-panc	*p* value^*^
	*n* = 91	*n* = 65	*n* = 19	*n* = 7	
**Age**					
years, mean (IQR)	68.9 (64-74)	69.1 (65-75)	69.2 (63-74)	65.0 (59-73)	0.41
**Sex**					
Female, n (%)	34 (37.4)	20 (30.8)	10 (52.6)	4 (57.1)	0.12
Male, n (%)	57 (62.6)	45 (69.2)	9 (47.4)	3 (42.9)	
**Location of IPMN**					
Head of the pancreas, n (%)	57 (62.6)	43 (66.2)	8 (42.1)	6 (85.7)	0.07
Pancreas body and tail, n (%)	34 (37.4)	22 (33.9)	11 (57.9)	1 (14.3)	
**MPD diameter**					
mm, median (IQR)	5.5 (3.1-8)	5.5 (3.1-8.7)	5.2 (2.8-7.6)	6.0 (3.6-7)	0.93
**Size of cyst**					
mm, median (IQR)	29 (20-40)	30 (20-44)	24 (18-38)	33 (16-42)	0.62
**Multifocal IPMN**					
yes, n (%)	23 (25.6)	15 (23.1)	7 (38.9)	1 (14.3)	0.31
**Clinical symptom**					
Jaundice, n (%)	5 (5.5)	0 (0)	1 (5.3)	4 (57.1)	< 0.001
Pancreatitis, n (%)	9 (9.9)	8 (12.3)	1 (5.3)	0 (0)	
none, n (%)	77 (84.6)	57 (87.7)	17 (89.5)	3 (42.9)	
**Enhancing solid component at CT**					
yes, n (%)	25 (27.5)	14 (21.5)	6 (31.6)	5 (71.4)	0.02
**Definite mural nodule at EUS**					
yes, n (%)	51 (59.3)	34 (55.7)	12 (63.2)	5 (83.3)	0.39
**MPD stenosis or disruption**					
yes, n (%)	17 (19.8)	6 (9.8)	8 (44.4)	3 (42.9)	0.002
**Pancreatic fluid cytology**					
Class 1, n (%)	1 (1.3)	0 (0)	1 (5.6)	0 (0)	< 0.001
Class 2, n (%)	32 (41.0)	27 (50.9)	5 (27.8)	0 (0)	
Class 3, n (%)	28 (35.9)	21 (39.6)	5 (27.8)	2 (28.6)	
Class 4, n (%)	3 (3.9)	2 (3.8)	0 (0)	1 (14.3)	
Class 5, n (%)	14 (20.0)	3 (5.7)	7 (38.9)	4 (57.1)	
**Serum CEA**					
ng/ml, median (IQR)	2.6 (1.8-4.0)	2.6 (1.7-4.2)	2.4 (1.7-3.1)	2.9 (2.4-8.1)	0.34
**Serum CA19-9**					
U/ml, median (IQR)	16.8 (8.6-41)	13.8 (8.2-30)	18.8 (12.4-63)	151 (83-155)	< 0.001
**Operative procedure**					
Pancreatoduodenectomy, n (%)	57 (62.6)	42 (64.6)	9 (47.4)	6 (85.7)	0.14
Middle pancreatectomy, n (%)	5 (5.5)	2 (3.1)	3 (15.8)	0 (0)	
Distal pancreatectomy, n (%)	29 (31.9)	21 (32.3)	7 (36.8)	1 (14.3)	
**Pathology**					
LGD, n (%)	42 (46.2)	38 (58.5)	4 (21.1)	0 (0)	< 0.001
HGD, n (%)	19 (20.9)	15 (23.1)	4 (21.1)	0 (0)	
IPMC, n (%)	30 (33.0)	12 (18.5)	11 (57.9)	7 (100)	
**Surgical margin**					
Negative, n (%)	60 (65.9)	47 (72.3)	9 (47.4)	4 (57.1)	0.005
LGD, n (%)	22 (24.2)	17 (26.2)	4 (21.1)	1 (14.3)	
HGD, n (%)	6 (6.6)	1 (1.5)	4 (21.1)	1 (14.3)	
IPMC, n (%)	3 (3.3)	0 (0)	2 (10.5)	1 (14.3)	
T factor of HGD/IPMC**					
Tis, n (%)	19 (38.8)	15 (55.6)	4 (26.7)	0 (0)	0.02
T1, n (%)	5 (10.2)	2 (7.4)	3 (20.0)	0 (0)	
T2, n (%)	2 (4.1)	2 (7.4)	0 (0)	0 (0)	
T3, n (%)	23 (46.9)	8 (29.6)	8 (53.3)	7 (100)	
**Lymph node metastasis in IPMC**^†^					
yes, n (%)	12 (25.0)	4 (33.3)	3 (27.3)	5 (71.4)	0.146
no, n (%)	18 (60.0)	8 (66.7)	8 (72.7)	2 (28.6)	
**Adjuvant chemotherapy for IPMC**^†^					
yes, n (%)	20 (66.7)	6 (50.0)	8 (72.7)	6 (85.7)	0.243
no, n (%)	10 (33.3)	6 (50.0)	3 (27.3)	1 (14.3)	

*Abbreviations*: *CA19-9* carbohydrate antigen 19-9, *CEA* carcinoembryonic antigen, *EUS* endoscopic ultrasonography, *HGD* high-grade dysplasia (non-invasive IPMN), *IPMC* IPMN with an associated invasive carcinoma, *IQR* interquartile range, *LGD* low- and intermediate-grade dysplasia, *MPD* main pancreatic duct

^*^Comparison between no recurrence, Rem-Panc, and Ex-Panc recurrence

^**^Counting for HGD and IPMC

^†^Counting for IPMC

**Fig. 1:**
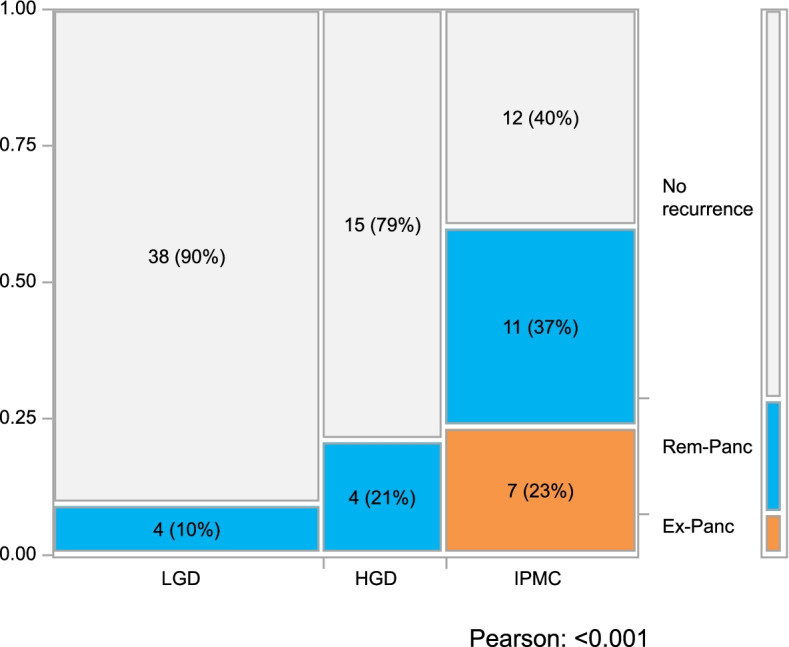
Correlations between the pathological grade of the resected IPMN lesion and recurrence patterns

Ex-Panc recurrence was characterized by a higher CA19-9 level and higher rates of clinical symptoms, enhancing solid component, class 4/5 on pancreatic juice cytology, and lymph node metastasis than Rem-Panc recurrence. Ex-Panc recurrence was observed only in IPMC patients as the primary pathology (*n* = 7, 23.3%). Distant metastatic sites in Ex-Panc recurrence were liver (*n* = 1), lung (*n* = 2), lymph nodes (*n* = 2), and peritoneum (*n* = 2). On the other hand, from the viewpoint of primary pathology, the frequency of Rem-Panc recurrence increased with pathological malignant grade; Rem-Panc recurrence occurred the most frequently in IPMC (*n* = 12, 36.7%), followed by HGD (*n* = 15, 21.1%) and LGD (*n* = 38, 9.5%) (Fig. 1). Regarding the relation between surgical margin and Rem-Panc recurrence, a positive surgical margin defined as IPMC or HGD was seen in six patients, of which four patients resulted in Rem-Panc recurrence at the distant site from the surgical margin.

**Fig. 2:**
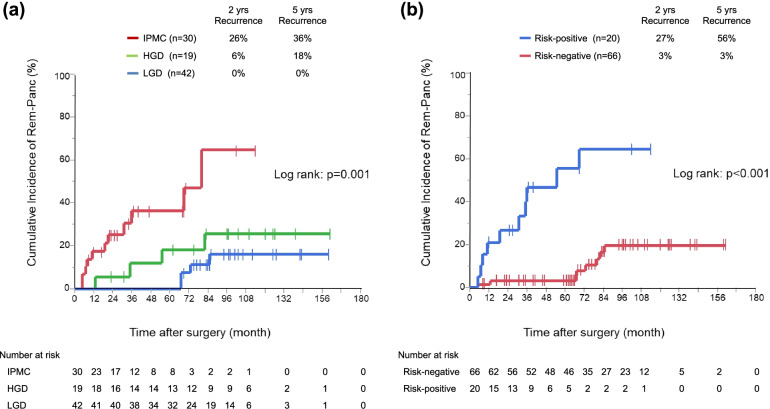
**a** Cumulative recurrence rate at the remnant pancreas, stratified by primary tumor grading (*n* = 91). **b** Cumulative recurrence rate at the remnant pancreas, stratified by risk factor (*n* = 86)

Concerning recurrence and survival analysis, median and mean follow-up periods were 71 months and 72.7 months, respectively. IPMC had the shortest recurrence-free time, followed by HGD and LGD; 2/5/10 year recurrence rates were 26/36/65% in IPMC, 6/18/26% in HGD, 0/0/16% in LGD, respectively (*p* = 0.001) (Fig. 2a). Multivariate analysis using perioperative parameters identified main duct stenosis or disruption (HR 10.63, *p* = 0.002) and HGD/IPMC at the surgical margin (HR4.4, *p* = 0.018) as independent risk factors for Rem-Panc recurrence (Table 2). Patients with one of these risk factors showed a significant potential for Rem-Panc recurrence compared to patients with no risk factors (2−/5−/10- year recurrence rates 27/56/64% vs. 3/3/20%, *p* < 0.001) (Fig. 2b).

**Table 2:** Univariate and multivariate analyses to examine risk factors for remnant pancreatic recurrence

Variables		Number	Univariate analysis			Multivariate analysis		
			Hazard ratio	95% CI	*p*-value	Hazard ratio	95% CI	*p*-value
**Background factor**								
Age (years)	≥65 vs < 65	68 vs 23	1.05	0.40-3.27	0.919	–	–	–
Sex	Male vs Female	57 vs 34	0.51	0.20-1.26	0.144	–	–	–
**Preoperative factor**								
Location of IPMN	pancreas head vs pancreas body/tail	57 vs 34	0.48	0.19-1.19	0.114	–	–	–
Multifocal IPMN	vs single lesion	23 vs 67	1.80	0.66-4.56	0.239	–	–	–
Obstructive jaundice	present vs absent	5 vs 86	4.15	0.22-24.39	0.266	–	–	–
Enhancing solid component on CT	present vs absent	25 vs 66	1.88	0.66-4.83	0.225	–	–	–
Main pancreatic duct (mm)	≥ 10 vs < 10	16 vs 75	0.82	0.19-2.46	0.743	–	–	–
Size of BD-IPMN (mm)	≥ 30 vs < 30	36 vs 39	0.51	0.14-1.57	0.247	–	–	–
Definite mural nodule on EUS	present vs absent	51 vs 35	1.21	0.49-3.26	0.682	–	–	–
MPD stenosis or disruption	present vs absent	17 vs 69	8.03	2.92-21.54	<.001	10.63	2.40-45.48	0.002
Pancreatic fluid cytology	class4-5 vs 1-3	17 vs 61	4.20	1.52-10.87	0.007	0.36	0.08-1.56	0.169
CEA (ng/ml)	elevated vs not elevated	24 vs 67	0.48	0.11-1.45	0.211	–	–	–
CA19-9 (U/ml)	elevated vs not elevated	24 vs 67	1.62	0.52-4.27	0.380	–	–	–
**Operative factor**								
Operative procedure	pancreatoduodenectomy vs distal/middle pancreatectomy	57 vs 34	0.58	0.23-1.45	0.243	–	–	–
Surgical margin	IPMC/HGD vs negative/LGD	9 vs 82	7.00	2.48-18.27	<.001	4.40	1.31-14.60	0.018
Pathological classification	IPMC vs LGD	30 vs 42	9.05	3.00-33.42	<.001	2.39	0.57-10.82	0.235
	HGD vs LGD	19 vs 42	2.18	0.51-9.22	0.280	2.18	0.47-9.94	0.308
	IPMC vs HGD	30 vs 19	4.15	1.36-15.46	0.011	1.09	0.31-4.49	0.891

*Abbreviations*: *CA19-9* carbohydrate antigen 19-9, *CEA* carcinoembryonic antigen, *HGD* high-grade dysplasia (non-invasive IPMN), *IPMC* IPMN with an associated invasive carcinoma, *LGD* low-grade and intermediate-grade dysplasia

Regarding the possible effect of adjuvant chemotherapy, of 30 patients diagnosed with IPMC, eight patients developed a Rem-Panc recurrence, and six patients developed Ex-Panc recurrence, despite adjuvant chemotherapy (*p* = 0.243). Furthermore, Rem-Pac recurrence-free survivals in 30 patients of IPMC and 23 patients of UICC-stage 2A/2B revealed that adjuvant chemotherapy did not significantly impact Rem-Panc recurrence (Supplementary Fig. 2). However, tumor advancements of IPMC were heterogeneous between patients with or without adjuvant chemotherapy (Supplementary Table).

**Fig. 3:**
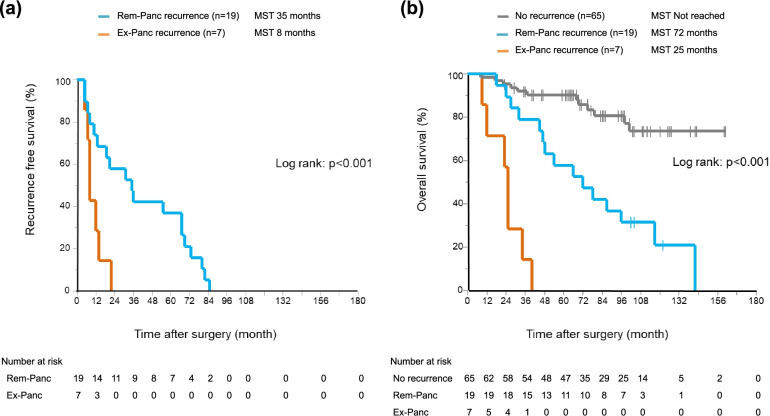
**a** Recurrence-free survival curves after resection, stratified by recurrence pattern (*n* = 26). **b** Overall-survival curves after resection, stratified by recurrence pattern (*n* = 91)

Concerning RFS and OS, Ex-Panc recurrence resulted in a worse prognosis than Rem-Panc recurrence (median RFS 8 months vs. 35 months, *p* < 0.0001; median OS 25 months vs. 72 months, *p* < 0.0001) (Fig. 3a, b).

**Fig. 4:**
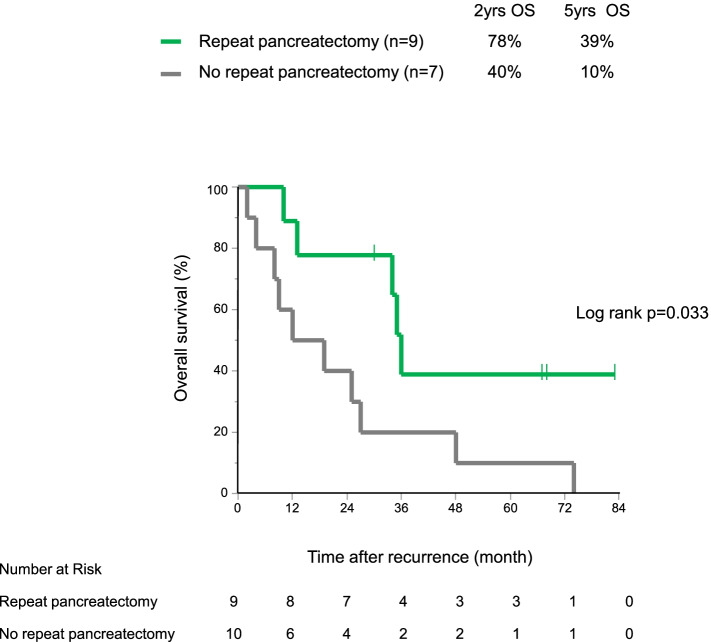
Overall survival curves after remnant pancreatic recurrence, comparing patients with and without repeat pancreatectomy (*n* = 19)

In patients with Rem-Panc recurrence (*n* = 19), repeat pancreatectomy was performed in 9 patients (47.3%). Survival after Rem-Panc recurrence of patients treated by repeat pancreatectomy was better than that of patients without any surgical intervention (median OS 36 months vs 15.5 months, *p* = 0.033) (Fig. 4).

## Discussion

International consensus guidelines suggest that there should be no time limit for follow-up after the resection of IPMNs, but the specific surveillance protocol remains unclear [3]. This study aimed to assess the clinical features of IPMN recurrence which could contribute to establishment of optimal surveillance protocols. RFS and OS differed between the recurrence patterns: Ex-Panc recurrence tended to occur earlier in the postoperative period and to resulted in a poorer prognosis than Rem-Panc recurrence. In the comparing of clinical background characteristics between patients with intrapancreatic or extrapancreatic recurrence, as shown in Fig. 2, all Ex-Panc recurrences occurred in primary IPMC cases. In addition, Ex-Panc recurrence showed a higher serum CA19-9 level, jaundice, and advanced UICC stage. Namely, Ex-Panc recurrence could rely heavily on primary tumor biology, and systemic screening during the early postoperative period would be preferable. Perhaps adjuvant chemotherapy or neoadjuvant chemotherapy should be considered in these cases.

On the other hand, Rem-Panc recurrence also depends on primary tumor biology. IPMC or HGD as primary pathology showed higher and earlier Rem-Panc recurrence than LGD. Furthermore, IPMC or HGD as a positive surgical margin could be an independent risk factor for Rem-Panc recurrence. Based on these results, the potential for ductal instability of Rem-Panc would be reflected in the degree of differentiation of the primary tumor. Furthermore, the fact that LGD showed Rem-Panc recurrence even after five years suggests that surveillance of Rem-Panc after resection of IPMN should be continued throughout the patient’s lifetime, regardless of tumor grading. Regarding post-Rem-Panc recurrence survival, only repeated surgery could be a curative treatment option that would contribute to long-term survival. The main reason why ten patients of Rem-Panc recurrence did not have the opportunity for repeat pancreatectomy was locally advanced Rem-Panc IPMC or ductal cell carcinoma with distant metastasis because of the delayed diagnosis of recurrent disease.

As the present results show, about half of the patients with the risk-positive disease develop Rem-Panc recurrence within five years after surgery, suggesting that repeated imaging checks each year, especially focusing on the pancreatic duct by MRCP, are recommended for early detection of Rem-Panc recurrence. Hirono reported recurrence patterns and risk factors after surgical resection of 1074 IPMNs as a project study of the Japan Pancreas Society [14]. This analysis classified recurrence types into “High-risk lesions in the remnant pancreas” and “Extrapancreatic recurrence”. Preoperative clinical symptoms, pancreatic body/tail as the IPMN location, main duct size > 10 mm, and HGD/invasive IPMC were identified as independent risk factors for “High-risk lesions in the remnant pancreas”. In addition to these findings, we first identified that preoperative EUS findings could be an essential predictor of Rem-Panc recurrence after IPMN resection, including HGD/IPMC at the surgical margin and main duct stenosis or disruption as a preoperative EUS feature. According to previous studies, there are contradictory opinions about the surgical margin. It has been reported that a positive margin is associated with the risk of postoperative recurrence [15, 16], whereas another paper showed there was no relationship [17, 18]. Interestingly, Frankel reported that dysplasia at the margin after pancreatectomy for non-invasive IPMN is associated with recurrence in the remnant gland [19]. In their analysis, 85% of Rem-Panc recurrence occurred at distant sites of Rem-panc epithelium from the surgical margin. They considered that when dysplasia is present at multiple locations within the pancreas, such as the surgical margin and/or extra-cystic duct, patients are at increased risk of developing recurrent IPMN, supporting the concept of a ‘field defect’. Similarly, 67% (4/6) of positive surgical margin resulted in Rem-Panc recurrence at the distant site from the surgical margin in our subject. Thus, IPMNs with positive margins would mean that pancreatic ductal epithelium with high carcinogenic potential is present in the entire remaining pancreas, and the residual pancreas is prone to recurrence. Moreover, Rem-Panc recurrence could occur not only at the actual surgical margin but at distant sights of Rem-Panc epithelium from the margin. Hence, it is suggested that a positive margin is more likely a marker of diffuse ductal instability rather than a local oncologic failure.

Knowledge about the correlation between recurrence risk and stenosis/disruption of the main pancreatic duct has been insufficient. Pea advocated three different mechanisms to explain the development of malignant lesions in the residual pancreas after IPMN surgery [20]: (1) tumor resection at the surgical margin of the pancreas; (2) spread of tumor cells into pancreatic ducts or parenchyma; and (3) independent multifocal lesions. The features of the main pancreatic duct might suggest tumor spread into remnant pancreatic parenchyma as in the second mechanism. In general, most studies did not report any significant difference in the risk of obstructive pancreatitis between benign and malignant IPMNs [21–23] because mucous embolism of the main pancreatic duct is more frequent in intestinal type with a better prognosis [24, 25]. In addition, the maximum diameter of the main pancreatic duct was correlated with the distance of tumor spread in the main pancreatic duct [26].

Consequently, a specific surveillance protocol for IPMN should be stratified, focusing on two different types of recurrence since half of the patients with a high risk of Rem-Panc recurrence would recur within five years after surgery. Therefore, Rem-Panc patients should be closely checked by various diagnostic modalities, including EUS or MRCP [27, 28]. Even in patients without any risk factors, the possibility of Rem-Panc recurrence persists throughout the patients’ lifetime; thus, uninterrupted surveillance is necessary. On the other hand, Ex-Panc recurrence was characterized only by IPMC as the primary lesion and would recur within two years. Therefore, careful follow-up 3-4 times a year for two years after surgery is desirable, and the selection of neo-adjuvant and adjuvant chemotherapy in addition to surgery is an issue for further study.

Practically, the following surveillance protocol according to the grade of IPMN could be suggested. In cases of IPMC, whole-body CT should be performed every three months for the first two years, alternating CT and MRCP to Rem-Panc for 3-5 years, and twice-yearly MRCP focusing on Rem-Panc for life after the 5th year. On the other hand, in cases of HGD or LGD, MRCP for Rem-Panc should be performed twice a year and once a year, respectively, for a lifetime. Moreover, in cases with some risks of Rem-Panc recurrence, surveillance should be enhanced more closely for the first five years and include proactive use of EUS.

Some limitations of our study were that it was a retrospective study at a single center, and the surveillance strategy was not standardized throughout the follow-up period. And though adjuvant chemotherapy for IPMC is generally accepted, it seemed not to improve Rem-Panc recurrence in our patients’ cohort. However, it should also be noted that the small size of the subjects and the likelihood of bias and heterogeneity made it difficult to analyze various aspects with great statistical power. It would be appropriate to address this topic explicitly in the future. Nonetheless, despite these limitations, it could be worthwhile to suggest the follow-up protocol based on the risk factors for Rem-Panc or Ex-Panc recurrence.

## Conclusions

The present study showed the possibility of IPMN stratification by risk according to a type of relapse using perioperative factors for the treatment strategy. IPMC cases might need systemic surveillance focusing on EX-Panc recurrence within two years after primary pancreatectomy. On the other hand, in any type of IPMN, remnant pancreatic screening is necessary for life, and interval screening depends on risk factors.

## Supplementary Information

**Additional file 1: Supplementary Figure 1.** Representative CT images of extra- and remnant pancreatic recurrence (a. liver metastasis; b. lung metastasis; c. peritoneal dissemination; d. solid component at remnant pancreas; e. main pancreatic duct dilatation with enhanced mural nodule).

**Additional file 2: Supplementary Figure 2.** (a) Cumulative recurrence rate at the remnant pancreas, stratified by adjuvant chemotherapy (IPMC, *n* = 30). (b) Cumulative recurrence rate at the remnant pancreas, stratified by adjuvant chemotherapy (UICC-stage 2A/2B, *n* = 23).

**Additional file 3: Supplementary Table.** Clinicopathological characteristics of patients diagnosed as IPMC (*n* = 30).

## Data Availability

The datasets generated and/or analyzed during the current study are not publicly available due owing to data privacy policy at our facility, but are available from the corresponding author on reasonable request.
