# Supplementary figures and images for: Optimal surveillance of intraductal papillary mucinous neoplasms of the pancreas focusing on remnant pancreas recurrence after surgical resection
Source: BMC Cancer. 2022 May 29;22:588. doi: 10.1186/s12885-022-09650-w (PMC9148522; doi:10.1186/s12885-022-09650-w)

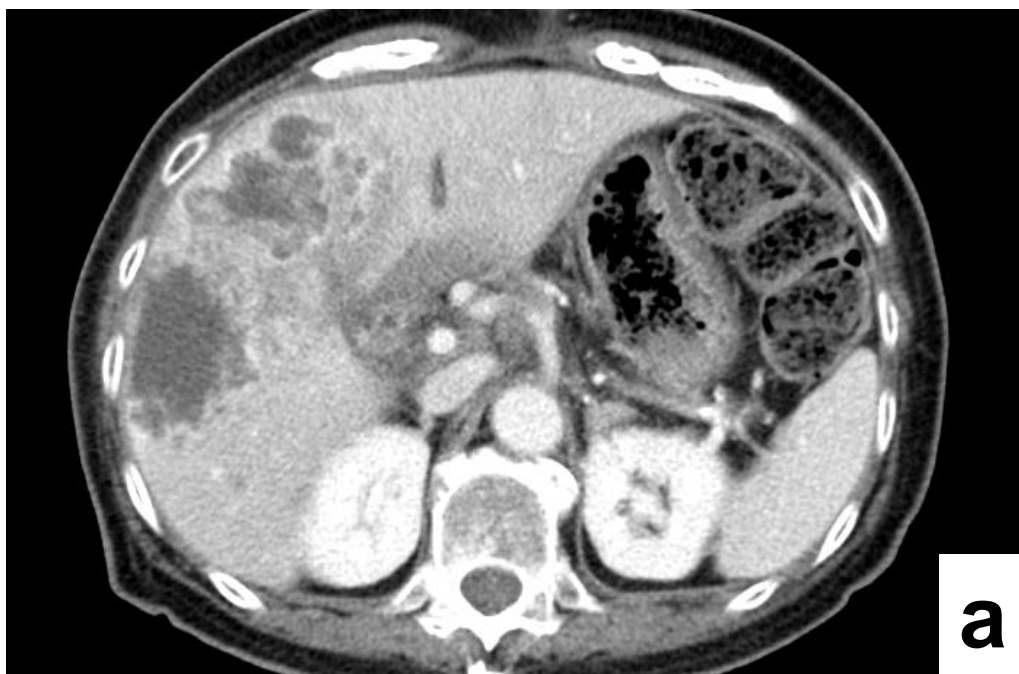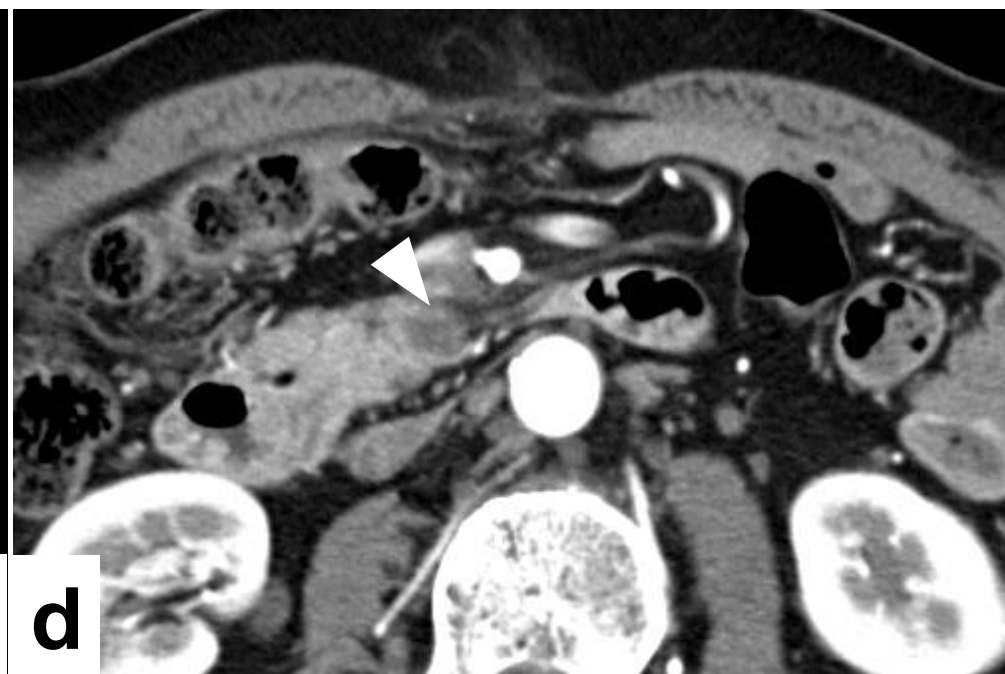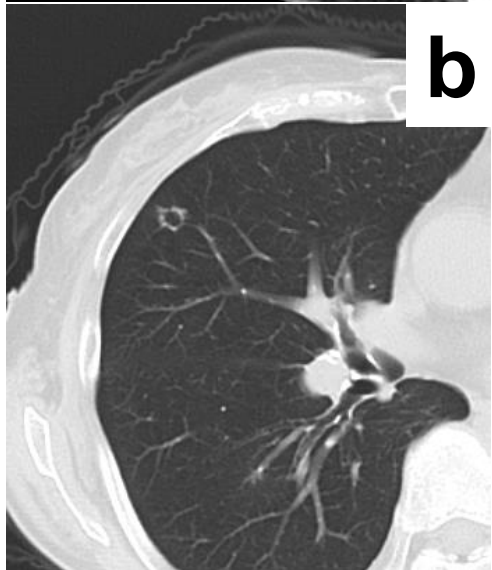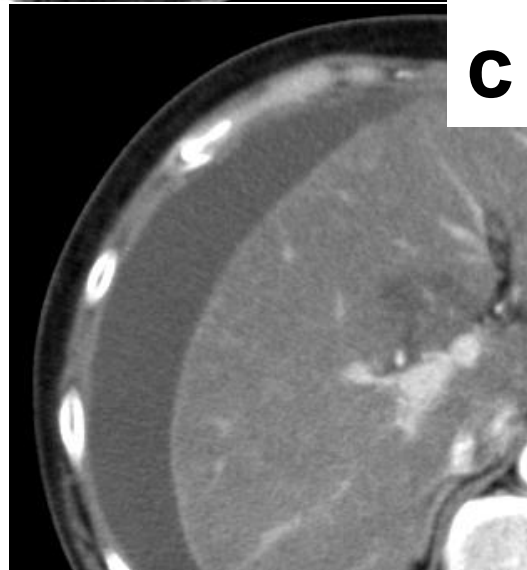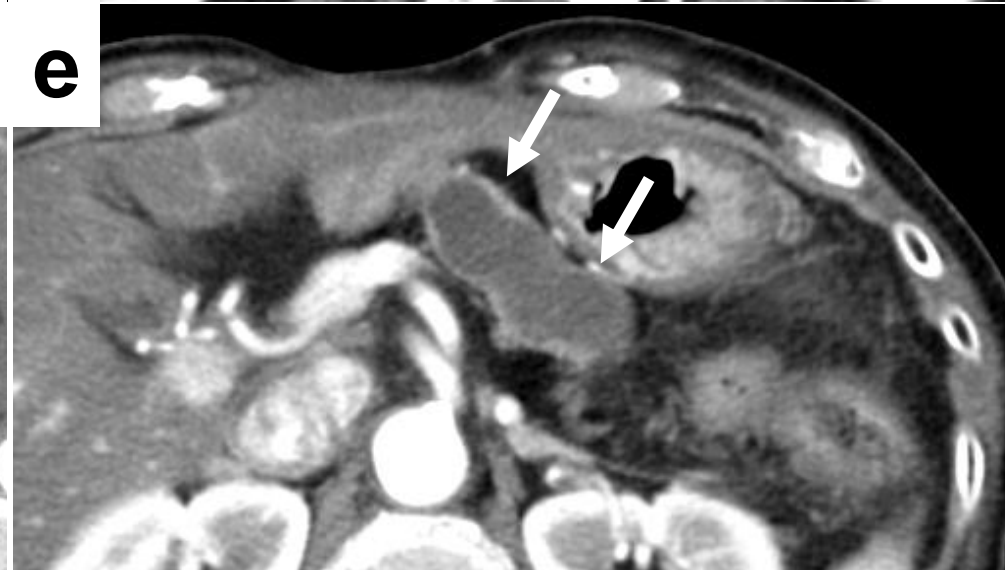

**a** **d**  
**c** **e**

Supplement: Supplementary file 1 — Additional file 1: Supplementary Figure 1. Representative CT images of extra- and remnant pancreatic recurrence (a. liver metastasis; b. lung metastasis; c. peritoneal dissemination; d. solid component at remnant pancreas; e. main pancreatic duct dilatation with enhanced mural nodule). [file 12885_2022_9650_MOESM1_ESM.pdf]
