# Supplementary material for: Optimal surveillance of intraductal papillary mucinous neoplasms of the pancreas focusing on remnant pancreas recurrence after surgical resection
Source: BMC Cancer. 2022 May 29;22:588. doi: 10.1186/s12885-022-09650-w (PMC9148522; doi:10.1186/s12885-022-09650-w)
Supplement: Supplementary file 2 — Additional file 2: Supplementary Figure 2. (a) Cumulative recurrence rate at the remnant pancreas, stratified by adjuvant chemotherapy (IPMC, n = 30). (b) Cumulative recurrence rate at the remnant pancreas, stratified by adjuvant chemotherapy (UICC-stage 2A/2B, n = 23). [file 12885_2022_9650_MOESM2_ESM.pdf]

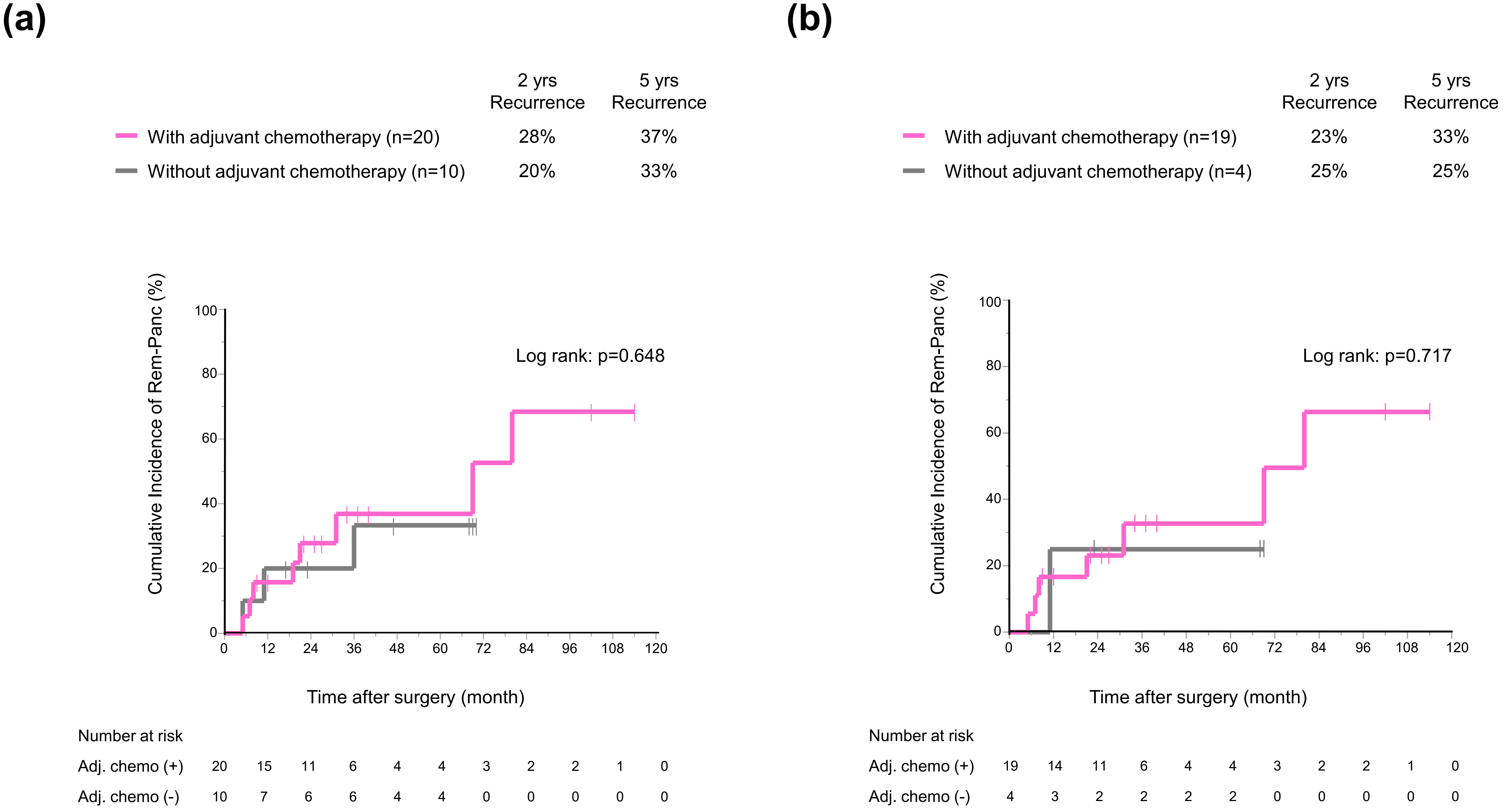
(a)
2 yrs Recurrence
5 yrs Recurrence
With adjuvant chemotherapy (n=20) 28% 37%
Without adjuvant chemotherapy (n=10) 20% 33%
Log rank: p=0.648
Cumulative Incidence of Rem-Panc (%)
0
20
40
60
80
100
0 12 24 36 48 60 72 84 96 108 120
Time after surgery (month)
Number at risk
Adj. chemo (+) 20 15 11 6 4 4 3 2 2 1 0
Adj. chemo (-) 10 7 6 6 4 4 0 0 0 0 0
(b)
2 yrs Recurrence
5 yrs Recurrence
With adjuvant chemotherapy (n=19) 23% 33%
Without adjuvant chemotherapy (n=4) 25% 25%
Log rank: p=0.717
Cumulative Incidence of Rem-Panc (%)
0
20
40
60
80
100
0 12 24 36 48 60 72 84 96 108 120
Time after surgery (month)
Number at risk
Adj. chemo (+) 19 14 11 6 4 4 3 2 2 1 0
Adj. chemo (-) 4 3 2 2 2 2 0 0 0 0 0
